# Presence of late gadolinium enhancement in "healthy subjects": correlation with chronic exposure to moderately high altitudes

**DOI:** 10.1186/1532-429X-18-S1-P280

**Published:** 2016-01-27

**Authors:** Lilia M Sierra-Galan, Maria-Elena Soto-Lopez, Victor A Ferrari, Yuchi Han

**Affiliations:** 1 grid.413678.fCardiology, American British Cowdray Medical Center, Mexico, D.F Mexico; 2 Rheumathology, National Institute of Cardiology "Ignacio Chavez", Mexico, Mexico; 3 grid.25879.310000000419368972Cardiovascular Division, Department of Medicine, Perelman School of Medicine, University of Pennsylvania, Philadelphia, PA USA; 4 grid.411115.10000000404350884Cardiovascular Imaging, Hospital of the University of Pennsylvania, Philadelphia, PA USA

## Background

Cardiac structural and functional changes due to chronic exposure to moderately high altitudes (HA) have been poorly described. There are many larger cities with altitudes above 2000 meters over sea level (OSL) where this information is relevant. The purpose of our investigation was to identify whether chronic hemodynamic changes and tissue abnormalities were present in those native residents of moderately HA in Mexico City varying from 2250 to 2760 meters OSL.

## Methods

We designed a retrospective case-control study between healthy native residents of Mexico City (Case group) vs. a sea level Control group from Boston, Massachusetts. The retrospective review was approved by both centers. We matched cohorts of healthy subjects with no traditional cardiovascular risks factors referred for evaluation of chest pain, palpitations, or dyspnea. Sea level controls also included healthy volunteer subjects. Studies were acquired on 1.5 T scanners (Achieva, Philips, NL) with similar CMR protocols using 0.2 mmol/kg of Magnevist (Bayer Health Care, NJ). Case group subjects had echocardiograms, which were reviewed. We analyzed RV and LV volumetric, functional, and late gadolinium enhanced (LGE) data. Statistical analysis was performed using student T test, ANOVA, univariate and multivariate linear and logistic regressions using SPSS version 12.0 (SPSS, Inc., Chicago, IL).

## Results

**Figure 1:**
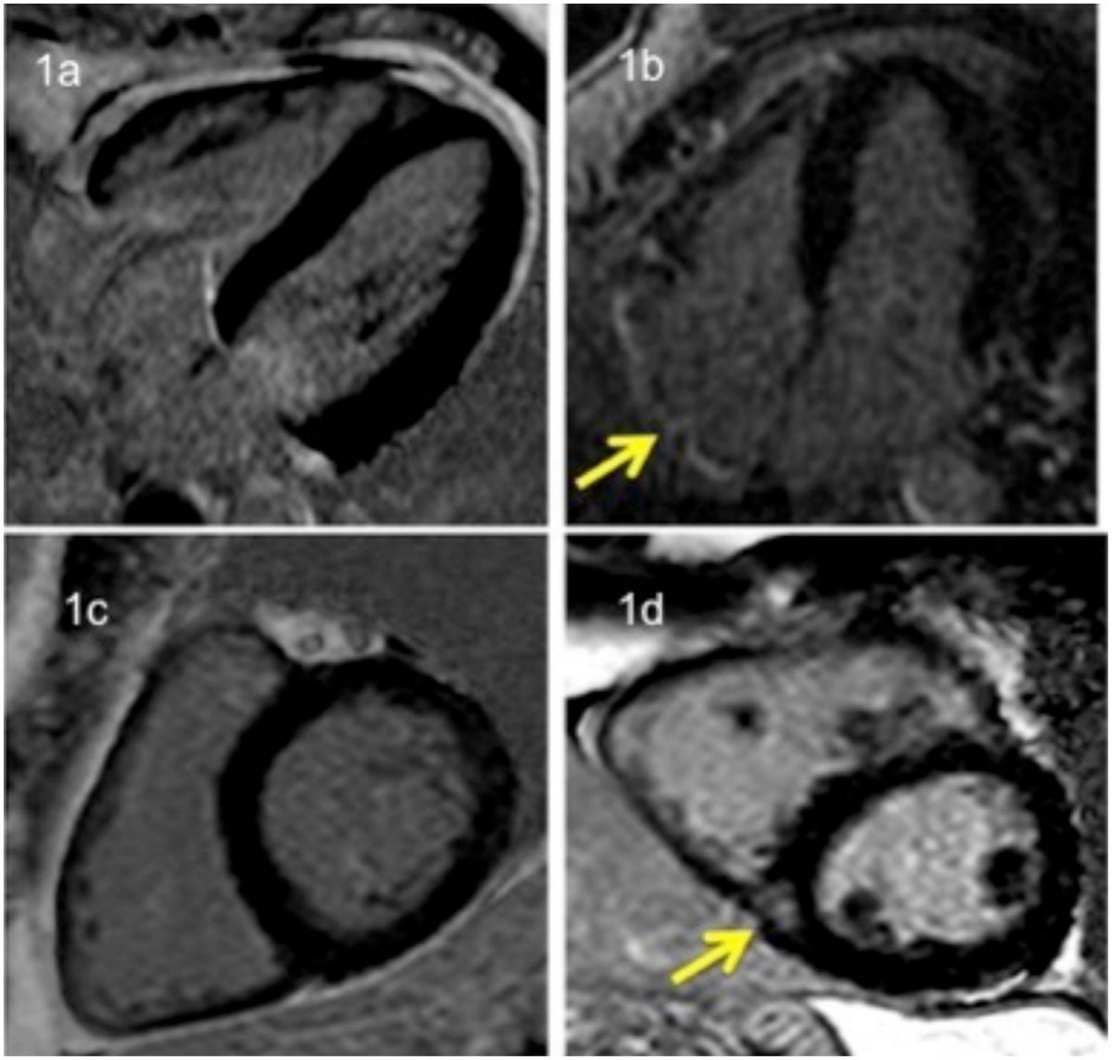
**LGE images**. Superior panels shows 4 chambers views, 1a shows a case with no LGE in RA, 1b shows a case with RA LGE (arrow). Inferior panels shows short axis views, 1c shows a case with no RV insertion point LGE, 1d shows a case with RV insertion LGE (arrow).

A retrospective cohort of 61 subjects was studied: 34 Controls (mean altitude of 3 meters OSL) and 27 Case subjects (mean altitude of 2480 + 245 meters OSL); 31 (51%) were male, mean age 39 ± 17 years and Controls were younger (33 ± 14 years) compared to Cases (45 ± 17 years), p= 0.004. BMI was similar in both groups. Estimated pulmonary artery systolic pressure (PASP) from cases was 26 + 3.5 mmHg. LV and RV EF% were normal and similar in both groups. Right ventricular end diastolic volume index (RVEDVi) showed differences between Cases and Controls, but may be related to age and gender (p < 0.0001, p = 0.009). All other RV volumes and all LV volumes were aligned with age, and gender differences in both groups and did not show significance after statistical adjustment. For LGE analysis one case was excluded because of the loss of LGE data. RV insertion point LGE was seen in 24 (92%) of Cases and none of Controls. Right atrial (RA) LGE was seen in 12 (46%) of Cases and none of Controls. The presence of RV insertion point and RA LGE correlates with age and altitude (for age [p=0.02, p < 0.0001], for altitude [p < 0.0001, p = 0.007]) and both patterns of LGE correlate with one another (p=0.002). Figure 1 shows LGE findings.

## Conclusions

Chronic exposure to moderately HA is associated with LGE in otherwise healthy subjects at the RV insertion point and in the RA. This finding is not associated with changes in RV volume and PASP. Our findings have not been previously reported, may have clinical impact, and need proof in a larger prospective study.

